# Long-term impact of COVID-19-related nonpharmaceutical interventions on tuberculosis: an interrupted time series analysis using Bayesian method

**DOI:** 10.7189/jogh.15.04012

**Published:** 2025-01-24

**Authors:** Yongbin Wang, Yue Xi, Yanyan Li, Peiping Zhou, Chunjie Xu

**Affiliations:** 1 Department of Epidemiology and Health Statistics, School of Public Health, Xinxiang Medical University, Xinxiang, China; 2 Beijing Key Laboratory of Antimicrobial Agents/Laboratory of Pharmacology, Institute of Medicinal Biotechnology, Chinese Academy of Medical Sciences & Peking Union Medical College, Beijing, China

## Abstract

**Background:**

The implementation of non-pharmaceutical interventions (NPIs) during the COVID-19 pandemic may inadvertently influence the epidemiology of tuberculosis (TB). (TB). However, few studies have explored how NPIs impact the long-term epidemiological trends of TB. We aimed to estimate the impact of NPIs implemented against COVID-19 on the medium- and long-term TB epidemics and to forecast the epidemiological trend of TB in Henan.

**Methods:**

We first collected monthly TB case data from January 2013 to September 2022, after which we used the data from January 2013 to December 2021 as a training data set to fit the Bayesian structural time series (BSTS) model and the remaining data as a testing data set to validate the model's predictive accuracy. We then conducted an intervention analysis using the BSTS model to evaluate the impact of the COVID-19 pandemic on TB epidemics and to project trends for the upcoming years.

**Results:**

A total of 590 455 TB cases were notified from January 2013 to September 2022, resulting in an annual incidence rate of 57.4 cases per 100 000 population, with a monthly average of 5047 cases (5.35 cases per 100 000 population). The trend in TB incidence showed a significant decrease during the study period, with an annual average percentage change of −7.3% (95% confidence interval (CI) = −8.4, −6.1). The BSTS model indicated an average monthly reduction of 25% (95% CI = 17, 32) in TB case notifications from January 2020 to December 2021 due to COVID-19 (probability of causal effect = 99.80%, *P* = 0.002). The mean absolute percentage error in the forecast set was 14.86%, indicating relatively high predictive accuracy of the model. Furthermore, TB cases were projected to total 43 584 (95% CI = 29 471, 57 291) from October 2022 to December 2023, indicating a continued downward trend.

**Conclusions:**

COVID-19 has had medium- and long-term impacts on TB epidemics, while the overall trend of TB incidence in Henan is generally declining. The BSTS model can be an effective option for accurately predicting the epidemic patterns of TB, and its results can provide valuable technical support for the development of prevention and control strategies.

The COVID-19 pandemic, which emerged in late 2019, has significantly threatened global public health and has led to unprecedented disruptions in health care systems worldwide [1]. Various nonpharmaceutical interventions (NPIs) were swiftly implemented to curb its spread, ranging from social distancing measures to lockdowns and travel restrictions. While they were crucial for controlling its transmission, these interventions have had unintended consequences on other infectious diseases, raising concerns among public health experts [2].

Tuberculosis (TB) is a highly contagious airborne disease caused by *Mycobacterium tuberculosis*, primarily transmitted through respiratory droplets and capable of diminishing the host’s immune system [3]. It has long been a major global health concern, with millions of new cases and deaths reported each year. The Global TB Report 2023 showed that the disease remained the world’s second leading cause of death from a single infectious agent in 2022, after COVID-19; about 10.3 million people developed TB in 2022 alone, with an estimated incidence rate of 133 per 100 000 persons and 1.30 million deaths [3]. The implementation of NPIs to combat COVID-19 has inadvertently disrupted TB prevention and control efforts, leading to delays in diagnosis, treatment, and care for TB patients [4]. Yet while recent studies have observed a reduction in TB morbidity [4–6], they have primarily focussed on the short-term effect of COVID-19-related NPIs in 2020 [4]. The NPIs were continued until 2023, albeit with varying intensity across countries. As a result, there is growing interest in understanding the medium- and long-term impacts of COVID-19-related NPIs on TB incidence.

Interrupted time series (ITS) analyses are crucial for assessing the effectiveness of interventions, policies, or programmes by enabling the determination of causality [7]. By comparing the trends in a specific outcome variable before and after an intervention, researchers can estimate its impact while accounting for pre-existing trends and potential confounding variables [7]. Among such analyses, the Bayesian structural time series (BSTS) model has emerged as a robust alternative to traditional approaches like autoregressive integrated moving average (ARIMA) and segmented regression models [8,9], which assume linear relationships and stationary data. The BSTS model allows for nonlinear trends, seasonality, and structural breaks to be incorporated into the analysis thanks to its flexibility, Bayesian framework, probabilistic forecasting capabilities, and interpretability, making it a valuable tool for analysing complex time series data and drawing robust conclusions about the causal effects of interventions over time [10].

The World Health Organization (WHO) has set ambitious targets for the eradication of TB, aiming for a 50% reduction in the incidence rate of new cases by 2025 and a further 90% reduction by 2035 [11]. There is currently a gap in research regarding the medium- and long-term effects of COVID-19-related NPIs on TB. We aimed to address this gap by quantitatively evaluating the impact of COVID-19-related NPIs on TB and by projecting TB incidence trends through a BSTS model. Our findings could inform policymakers in optimising resource allocation and prioritising strategies for enhancing TB control efforts.

## METHODS

We retrieved monthly case reports of TB and COVID-19 between January 2013 and September 2022 from the Notifiable disease of Henan Provincial Health Committee [12] and the total population from the statistical yearbook of Henan [13]. The diagnostic criteria for TB were divided into suspected cases, clinically diagnosed cases, and confirmed cases, as outlined below.

**Suspected cases:**
− adults and children over five years of age who only have active TB lesions on chest imaging:
− children under five years of age who have suspected symptoms of TB, accompanied by either a history of close contact with smear-positive TB patients, moderately positive Mantoux test, or a γ-positive interferon release test.

**Clinical diagnosis cases:**
− adults with active TB lesions on chest imaging, accompanied by either suspicious symptoms of TB, a moderately positive Mantoux test, a γ-positive interferon release test, a positive TB antibody test, extrapulmonary histopathology confirmed as TB lesions, or a bronchoscopy consistent with the change of TB.
− children with active TB on chest imaging, suspicious symptoms of TB, moderately positive Mantoux test, or above γ-positive interferon release test.

**Confirmed cases:**
− positive sputum smear;
− positive for only *Mycobacterium* isolation and culture;
− positive in molecular biology;
− positive for pathology of TB.

The case reports contained all three types of cases diagnosed by various levels and types of medical and health institutions. As it is essential to promptly report an epidemic of TB patients, suspected patients, or pathogen carriers through the infectious disease epidemic monitoring information system within 24 hours, prevention and control institutions, along with medical facilities, conduct daily checks on reported information and eliminate any duplicate entries.

For our analysis, we gathered a data set spanning nearly 10 years. Typically, a minimum of 50 observed values is required to develop an effective predictive model [14]. We therefore used the TB data from January 2013 to December 2021 as the training data set to showcase the predictive potential of the BSTS method and the data from January 2022 to September 2022 as the test data set to validate its predictive performance, demonstrating its applicability and adequacy in estimating the epidemiological trend of TB incidence.

### BSTS model

The BSTS model’s prediction relies on prior information and likelihood functions which are combined to create a posterior distribution. The Markov Chain Monte Carlo (MCMC) sampling algorithm is employed to estimate the posterior distribution of each parameter of the model [10]. By utilising Bayesian model averaging, predictions based on posterior distributions can be smoothed, after which the final estimate is generated by averaging the results [15,16].

Unlike traditional linear models, the BSTS model employs dynamic confidence intervals (CIs) to evaluate the evolving impact of differences between intrinsic and counterfactual observations [17]. It offers several advantages, such as incorporating prior information and complex covariate structures, making it superior to traditional models. While traditional statistical methods like static regression and ARIMA have been used in the past to predict disease epidemic patterns, their linear nature limits their applicability [17]. The BSTS model allows parameters to evolve over time, accommodating several predictors and accurately revealing the stochastic behaviour of time series data.

The BSTS model enables the incorporation of prior information on variables and facilitates the selection of suitable variables through spike and slab priors [10]. As a stochastic state-space model, it can separately investigate trends, seasonality, and regression components, allowing it to effectively capture the dynamic nature of TB incidence and the influence of COVID-19 [17]. By considering spike and slab priors for optimal covariate selection and using Bayesian model averaging for generating forecasts, the BSTS model offers robust and reliable predictions, especially in dynamic systems [17].

In traditional statistical methods, the assumption of stability in the trend, seasonality, and regression components of time series data often does not align with the dynamic nature of real-world systems [17]. This mismatch can lead to inaccuracies in predictions and modelling. These methods can also suffer from overfitting issues when incorporating regression factors [17]. The BSTS model, in turn, leverages Bayesian inference, which incorporates prior beliefs about the parameters into the modelling process [17]. By assigning prior distributions to the parameters, the BSTS model introduces regularisation, which helps prevent over-fitting by penalising overly complex models [17]. It further employs techniques such as MCMC sampling to estimate the posterior distribution of parameters, allowing for uncertainty quantification and model averaging [10]. This Bayesian approach not only provides a more robust estimation of parameters, but also helps in selecting simpler models that better capture the underlying structure of the time series data. Furthermore, the BSTS model allows for time-varying model parameters, this flexibility enables it to adapt to changes in the underlying data structure over time [10]. Importantly, the prediction outcomes of BSTS are robust and less dependent on specific assumptions [17], enhancing the reliability and stability of forecasts, particularly in intricate and dynamic systems like TB epidemiology.

The BSTS model is particularly well-suited for estimating the causal impact of interventions on diseases’ epidemics: by predicting counterfactual scenarios, it can reveal how the outcome of an intervention would differ if the intervention had not taken place [10]. This is crucial for understanding the true effects of interventions and external shocks on complex systems like disease epidemiology [18]. In summary, the adaptability of BSTS model to time-varying parameters, its probabilistic forecasting approach, its ability to capture nonlinear relationships, and its capacity for counterfactual analysis make it useful for investigating the impact of events like the COVID-19 pandemic on TB case notifications [10].

The intervention period in our study spanned from January 2020 to September 2022. The modelling approach involved estimating the parameters of the BSTS model using data up to December 2019, prior to the intervention period caused by the COVID-19 pandemic. We then used the model to predict TB incidence during the period unaffected by interventions. After forecasting the intervention-free period, we applied it to data from January 2020 to September 2022, thereby catching the impact of COVID-19 NPIs on TB transmission dynamics. By comparing the predicted TB incidence with the actual observations during this post-intervention period, we could quantify the causal impact of the COVID-19 pandemic on TB trends.

To predict TB incidence, we had to conduct multiple simulations with different data sets to identify the most suitable trend models that yield accurate and reliable predictions. After repeated attempts, we selected a semi-local linear trend model within the BSTS framework [10]. Using TB data from January 2013 to December 2021 for testing, the model forecasted TB incidence from January 2022 to September 2022. We evaluated the accuracy of these predictions using the mean absolute percentage error (MAPE) metric [19], demonstrating whether there was a good fit between predicted and actual values. To extend the forecasting horizon, we then re-simulated data from January 2013 to September 2022 to predict TB incidence from October 2022 to December 2023.

### Statistical process

We used Hodrick-Prescott (HP) technology to decompose the trend and cycle component in TB incidence [20], and we utilised the seasonal index (which helps in determining the relative strength or weakness of a particular season compared to the overall average [21]) to quantify the seasonal patterns of TB epidemics. We adopted the average annual percentage change (AAPC) and annual percentage change (APC) along with their CIs to investigate the change trends in TB incidence using Joinpoint software, version 4.9.1.0 (National Cancer Institute, Bethesda, Maryland, USA) [22]. We also used the ‘CausalImpact’ and ‘bsts’ packages in R, version 4.2.0 (R Core Team, Vienna, Austria) to quantify the causal impacts and to predict TB epidemic patterns, with 500 iterations for robustness. We utilised the MAPE to estimate the forecasting accuracy of the BSTS model. Typically, a MAPE≤20% is considered indicative of a model accurately capturing the epidemic pattern of a disease [23], thus enabling future TB data predictions.

When conducting causal effect analysis using the BSTS method, it is essential to construct a control time series that is not influenced by interventions to ensure the credibility of the findings. Furthermore, the BSTS method operates on the assumption that the relationship between predictors and the response metric established during the pre-period remains consistent throughout the post-period [4]. Early research has suggested that seasonal patterns of infectious diseases serve as significant predictors [4]. Moreover, the total population is a factor linked to reported cases and typically remains stable despite interventions. With this in mind, we considered covariates such as seasonality, time variable, and population data when examining the long-term impact of the COVID-19 outbreak on TB epidemic using the BSTS model [4].

## RESULTS

### Statistical description

**Figure 1:**
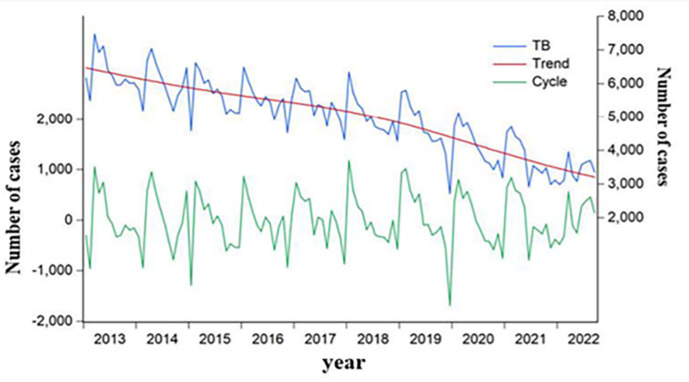
Trends and cycle patterns of TB epidemic situation in Henan from January 2013 to September 2022 (the restriction period of COVID-19 is January 2020).

**Figure 2:**
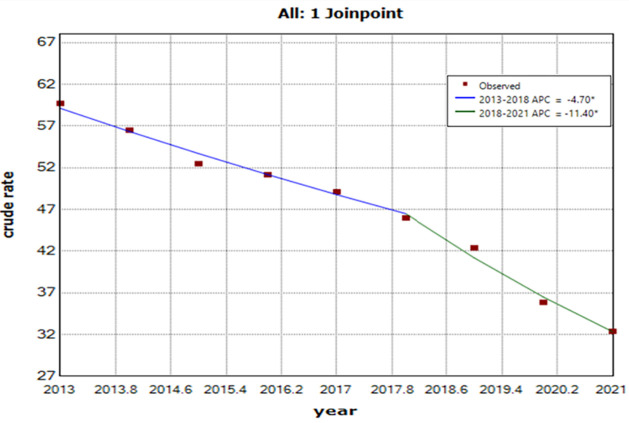
Joinpoint regression plot displaying the TB epidemiological trends from 2013–21. *APC is statistically significant.

A total of 590 455 TB cases were reported from January 2013 to September 2022, with an annual incidence rate of 57.4 cases per 100 000 population. The monthly average of notifications stood at 5047 (5.35 cases per 100 000 population) cases. The application of the HP decomposition method showed a significant decline in the overall epidemic trend of TB (**Figure 1**). We also observed seasonal variation in TB incidence; the monthly seasonal indices from January to December were 1.03, 1.07, 1.11, 1.08, 1.03, 0.99, 0.98, 0.92, 0.92, 0.96, 0.92, and 0.90, respectively, demonstrating higher rates in March-April and lower rates in November–December. The joinpoint regression analysis indicated an overall downward trend in TB incidence, with an AAPC of −7.3% (95% CI = −8.4, −6.1; *t* = −12.3; *P* < 0.001). However, the trend varied across different time intervals (**Figure 2**). The APC was −4.7 (95% CI = −6.2, −3.1; *t* = −8.2; *P* < 0.001) from 2013 to 2018, indicating a decrease, while there was a more pronounced decline from 2018 to 2021, with an APC of −11.4 (95% CI = −14.6, −8.1; *t* = −9.2; *P* < 0.001). This suggests a significant acceleration in the reduction of TB cases in the latter period compared to 2013–18.

### Impacts of COVID-19-associated interventions on TB

**Figure 3:**
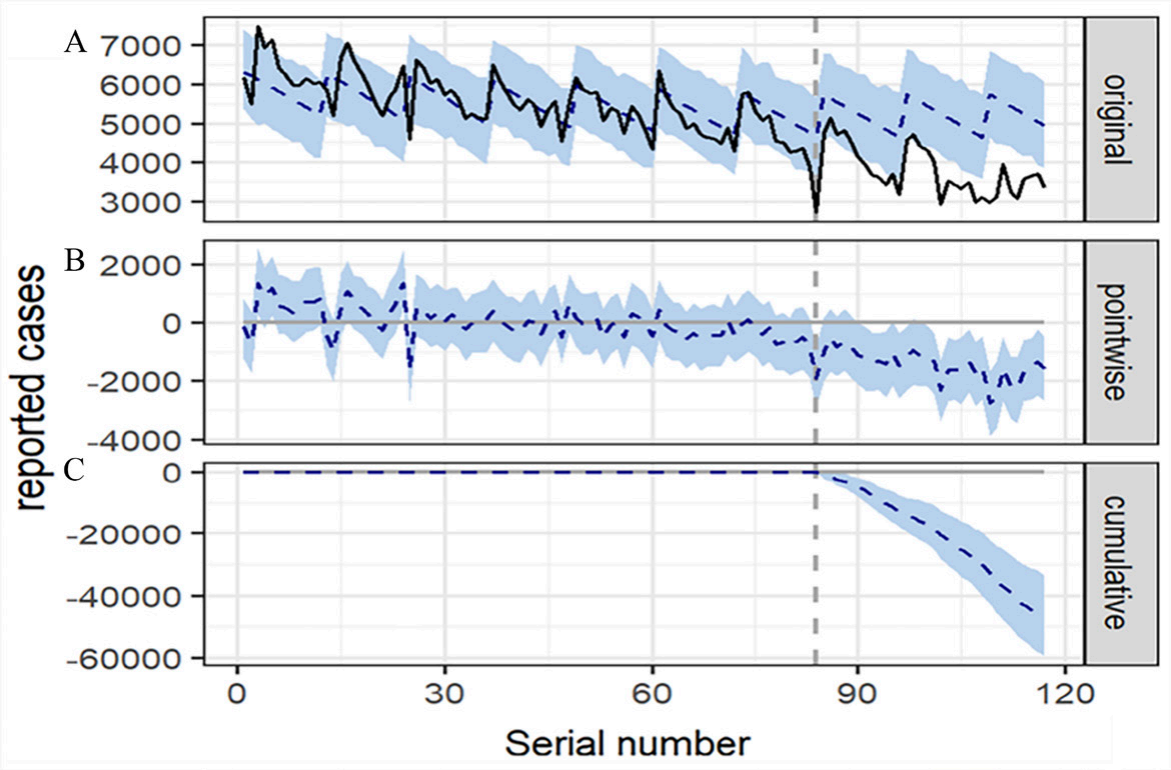
Time series plot displaying the causal impacts of the COVID-19 outbreak on the decreases in TB cases from January – September 2022. **Panel A.** Reported TB cases and counterfactual expected figures for the post-outbreak period. **Panel B.** Pointwise causal impact that indicates the difference between reported cases and expected figures. **Panel C.** Cumulative effect of the COVID-19 pandemic on the decreases in TB cases *via* accumulating the pointwise effects from the second panel.

Based on the predicted expected cases, the monthly average notification of TB cases decreased by 14% (95% CI = 1.6, 26) from January to March 2020, 15% (95% CI = 5.9, 24) from January to June 2020, and 20% (95% CI = 11, 30) from January to December 2020 as a result of the COVID-19 pandemic (**Figure 3**, **Table 1**). It is evident that COVID-19-related interventions played a role in reducing TB cases in 2020. Further, according to the BSTS analysis, the monthly average reduction in TB case notifications from January 2020 to June 2021 was 22% (95% CI = 13, 29), 25% (95% CI = 17, 32) from January 2020 to December 2021, and 28% (95% CI = 20, 34) from January 2020 to September 2022 due to COVID-19-related interventions (probability of causal effect = 99.80%; *P* = 0.002). These findings suggest that COVID-19-associated interventions may have had medium- and long-term implications for TB epidemics.

**Table 1:** Causal effects of the COVID-19 pandemic on the reductions in the monthly average and cumulative number of TB cases

Months	Average cases	Predictions (95% CI)	Absolute effect (95% CI)	Cumulative	Predictions (95% CI)	Absolute effect (95% CI)	Relative effect, % (95% CI)	*P*-value	Probability of causal effect, %
1	4739	5736 (4604, 6729)	−997 (−1990, 135)	4739	5736 (4604, 6729)	−997 (−1990, 135)	−17 (−35, 2.4)	0.0523	95.00
1–2	4935	5687 (4893, 6557)	−752 (−1622, 42)	9870	11 374 (9787, 13 115)	−1504 (−3245, 83)	−13 (−29, 0.73)	0.032	96.76
1–3	4864	5638 (4955, 6331)	−774 (−1467, −91)	14 591	16 913 (14 865, 18 992)	−2322 (−4401, −274)	−14 (−26, −1.6)	0.010	98.99
1–4	4852	5588 (4936, 6252)	−736 (−1400, −84)	19 409	22 353 (19 743, 25 008)	−2944 (−5599, −334)	−13 (−25, −1.5)	0.018	98.17
1–5	4784	5539 (4933, 6153)	−754 (−1368, −149)	23 922	27 693 (24 667, 30 763)	−3771 (−6841, −745)	−14 (−25, −2.7)	0.006	99.39
1–6	4678	5489 (5000, 6008)	−811 (−1329, −322)	28 071	32 934 (30 001, 36 046)	−4863 (−7975, −1930)	−15 (−24, −5.9)	0.008	99.19
1–7	4571	5440 (4858, 5932)	−868 (−1361, −287)	31 998	38 077 (34 007, 41 527)	−6079 (−9529, −2009)	−16 (−25, −5.3)	0.002	99.80
1–8	4460	5390 (4896, 5915)	−930 (−1455, −436)	35 680	43 120 (39 166, 47 321)	−7440 (−11 641, −3486)	−17 (−27, −8.1)	0.002	99.80
1–9	4367	5341 (4904, 5803)	−973 (−1435, −537)	39 307	48 066 (44 138, 52 226)	−8759 (−12 919, −4831)	−18 (−27, −10)	0.002	99.80
1–10	4274	5291 (4792, 5781)	−1018 (−1508, −518)	42 735	52 912 (47 918, 57 811)	−10 177 (−15 076, −5183)	−19 (−28, −9.8)	0.002	99.80
1–11	4222	5242 (4749, 5693)	−1020 (−1471, −527)	46 441	57 658 (52 240, 62 622)	−11 217 (−16 181, −5799)	−19 (−28, −10)	0.002	99.80
1–12	4135	5192 (4728, 5668)	−1057 (−1533, −593)	49 620	62 307 (56 741, 68 018)	−12 687 (−18 398, −7121)	−20 (−30, −11)	0.002	99.80
1–13	4167	5234 (4789, 5690)	−1067 (−1523, −621)	54 176	68 041 (62 255, 73 975)	−13 865 (−19 799, −8079)	−20 (−29, −12)	0.002	99.80
1–14	4206	5263 (4836, 5684)	−1057 (−1478, −631)	58 880	73 676 (67 709, 79 577)	−14 796 (−20 697, −8829)	−20 (−28, 12)	0.002	99.80
1–15	4219	5281 (4823, 56 5)	−1062 (−1466, −604)	63 287	79 213 (72 351, 85 281)	−15 926 (−21 994, −9064)	−20 (−28, −11)	0.002	99.80
1–16	4225	5355 (4904, 5744)	−1129 (−1519, −678)	67 607	85 676 (78 456, 91 904)	−18 069 (−24 297, −10 849)	−21 (−28, −13)	0.001	99.90
1–17	4213	5294 (4903, 5714)	−1080 (−1501, −690)	71 623	89 991 (83 346, 97 134)	−18 368 (−25 511, −11 723)	−20 (−28, −13)	0.002	99.80
1–18	4141	5291 (4855, 5687)	−1149 (−1546, −713)	74 544	95 232 (87 383, 102 373)	−20 688 (−27 829, −12 839)	−22 (−29, −13)	0.002	99.80
1–19	4110	5347 (4883, 5720)	−1237 (−1610, −773)	78 088	101 589 (92 778, 108 686)	−23 501 (−30 598, −14 690)	−23 (−30, −14)	0.001	99.90
1–20	4076	5271 (4851, 5677)	−1194 (−1601, −774)	81 528	105 416 (97 012, 113 547)	−23 888 (−32 019, −15 484)	−23 (−30, −15)	0.002	99.80
1–21	4041	5255 (4799, 5668)	−1215 (−1628, −758)	84 855	110 360 (100 770, 119 038)	−25 505 (−34 183, −15 915)	−23 (−31, −14)	0.002	99.80
1–22	4016	5300 (4868, 5648)	−1285 (−1632, −852)	88 342	116 608 (107 094, 124 253)	−28 266 (−35 911, −18 752)	−24 (−31, −16)	0.001	99.90
1–23	3971	5215 (4768, 5617)	−1245 (−1647, −798)	91 325	119 951 (109 672, 129 195)	−28 626 (−37 870, −18 347)	−24 (−32, −15)	0.002	99.80
1–24	3935	5255 (4814, 5627)	−1320 (−1692, −879)	94 439	126 124 (115 536, 135 049)	−31 685 (−40 610, −21 097)	−25 (−32, −17)	0.001	99.90
1–25	3897	5213 (4797, 5558)	−1316 (−1662, −900)	97 422	130 334 (119 916, 138 960)	−32 912 (−41 538, −22 494)	−25 (−32, −17)	0.002	99.80
1–26	3867	5230 (4790, 5573)	−1363 (−1706, −924)	100 536	135 970 (124 553, 144 889)	−35 434 (−44 353, −24 017)	−26 (−33, −18)	0.002	99.80
1–27	3870	5241 (4818, 5585)	−1371 (−1715, −948)	104 496	141 507 (130 091, 150 794)	−37 011 (−46 298, −25 595)	−26 (−33, −18)	0.002	99.80
1–28	3848	5248 (4821, 5610)	−1400 (−1761, −973)	107 758	146 945 (134 997, 157 072)	−39 187 (−49 314, −27 239)	−27 (−34, −19)	0.002	99.80
1–29	3822	5251 (4844, 5632)	−1429 (−1810, −1022)	110 836	152 286 (140 483, 163 319)	−41 450 (−52 483, −29 647)	−27 (−34, −19)	0.002	99.80
1–30	3814	5251 (4821, 5603)	−1437 (−1789, −1007)	114 411	157 529 (144 631, 168 094)	−43 118 (−53 683, −30 220)	−27 (−34, −19)	0.002	99.80
1–31	3809	5248 (4822, 5640)	−1439 (−1831, −1014)	118 065	162 673 (149 491, 174 839)	−44 608 (−56 774, −31 426)	−27 (−35, −19)	0.002	99.80
1–32	3805	5241 (4839, 5606)	−1436 (−1801, −1034)	121 763	167 718 (154 837, 179 407)	−45 955 (−57 644, −33 074)	−27 (−34, −20)	0.002	99.80
1–33	3791	5232 (4831, 5582)	−1441 (−1791, −1040)	125 115	172 664 (159 427, 184 216)	−47 549 (−59 101, −34 312)	−28 (−34, −20)	0.002	99.80

CI – confidence interval

By considering the Bayesian confidence intervals and the posterior probability of causal effect using BSTS (**Table 1**), we see that the posterior probabilities leading to these effects as random occurrences can be rejected, thus confirming the substantial evidence of the true effect [17].

### Nowcasting and forecasting the epidemiological trends of TB

The training data set utilised data from January 2013 to December 2021, with the BSTS model employed to forecast data from January 2022 to September 2022. We included a variable in the model to account for the removal of the COVID-19 effect, where a value of ‘0’ indicated pre-COVID-19 outbreak and ‘1’ post-outbreak. By averaging across 500 MCMC draws under the BSTS model, we generated forecasts and their 95% CI (**Table 2**). The predicted values for the testing data set closely aligned with the observed values, yielding a MAPE of 14.86%.

**Table 2:** Forecasted TB cases from January 2022 to September 2022 with the BSTS model

Months, 2022	Observed values	Forecasts	95% CI
January	2983	4241	3251, 5242
February	3114	3969	2975, 4921
March	3960	3948	2943, 4913
April	3262	3773	2826, 4738
May	3078	2980	1848, 3999
June	3575	3379	2342, 4434
July	3654	3097	2049, 4127
August	3698	3050	1973, 4194
September	3352	3127	2050, 4241

BSTS – Bayesian structure time series, CI – confidence interval

**Figure 4:**
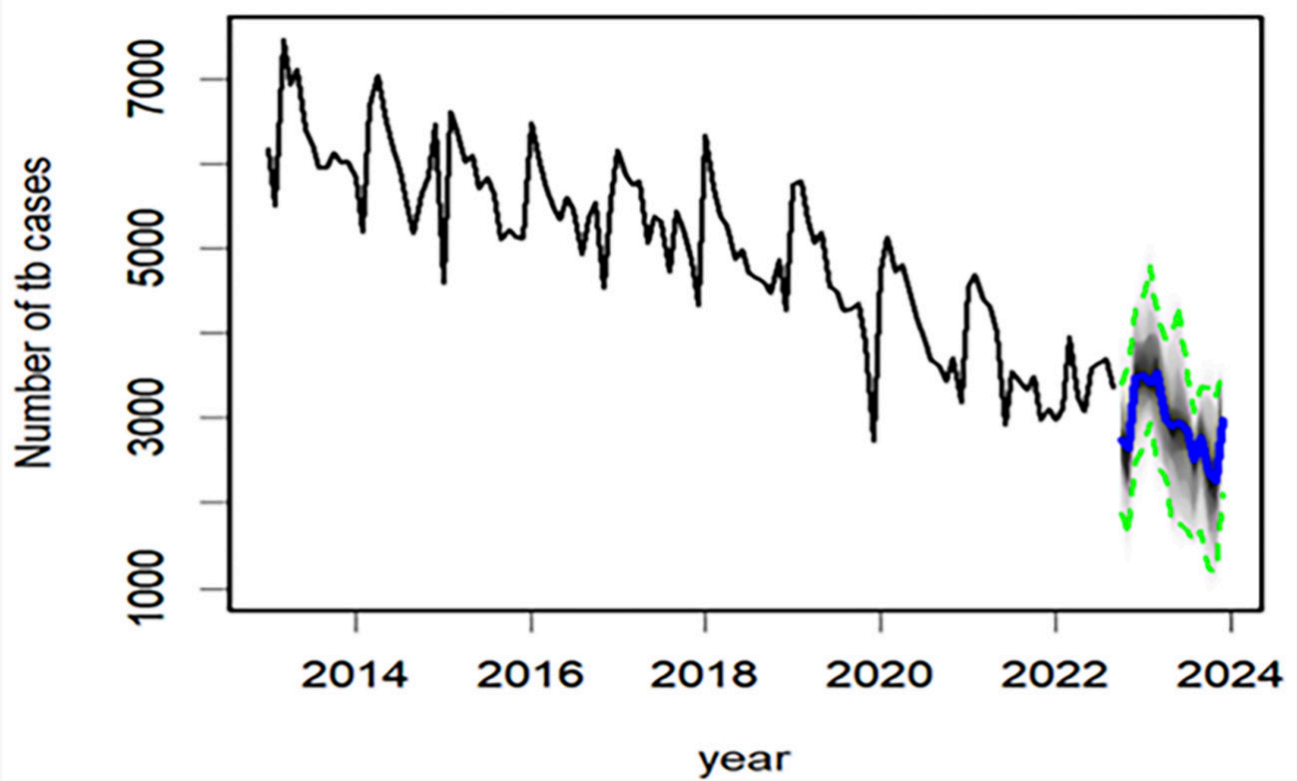
Incidence trend of TB in Henan from September 2022 to December 2023, as predicted by the BSTS model.

Subsequently, by re-fitting data from January 2013 to September 2022, we predicted the epidemic trend of TB between October 2022 and December 2023 (**Figure 4**, **Table 3**). We found that the total number of TB cases from October 2022 to December 2023 was estimated to be 43 584 (95% CI = 29 471, 57 291), with a monthly average of 2906 cases (95% CI = 1965, 3819). The trend in TB incidence may continue to exhibit a decline during this period.

**Table 3:** Predicted TB cases from October 2022 to December 2023 based on the BSTS model

Months, 2022–23	Forecasts	95% CI
October 2022	2720	1896, 3410
November 2022	2561	1645, 3633
December 2022	3458	2474, 4341
January 2023	3533	2680, 4433
February 2023	3580	2946, 4802
March 2023	3445	2470, 4248
April 2023	3030	2301, 3921
May 2023	2880	1739, 4013
June 2023	2899	1792, 4350
July 2023	2743	1717, 3497
August 2023	2428	1551, 3058
September 2023	2707	1693, 3381
October 2023	2321	1245, 3366
November 2023	2254	1211, 3224
December 2023	3023	2111, 3614

CI – confidence interval

## DISCUSSION

Accurate prediction of TB incidence plays a crucial role in the prevention and treatment. The BSTS model we used here leverages the analysis of temporal changes in TB incidence to forecast the future epidemic trajectory. As shown in our findings (**Table 2**), the predicted values closely matched the observed data, with a predictive MAPE value below 20% under the BSTS model, highlighting its efficacy. This type of model has been successfully applied in forecasting alcohol-related harms in England and assessing the causal effects of new local alcohol licensing policies on hospital admissions and crime [24]. It can therefore be recommended as an alternative tool for developing and executing intervention strategies to address adverse health outcomes in public health settings. Our analysis showed that the COVID-19 pandemic resulted in a significant decrease in the average monthly notification rate of TB by 22% (95% CI = 13, 29) from January 2020 to June 2021, 25% (95% CI = 17, 32) from January 2020 to December 2021, and 28% (95% CI = 20, 34) from January 2020 to September 2022 (causal impact probability = 99.80%, *P* = 0.002). This indicated a downward trend in TB incidence from January 2020 to September 2022, suggesting that the stringent measures implemented in response to COVID-19 have significantly reduced TB notifications in Henan, reflecting the effectiveness of various measures taken to combat COVID-19. The robust response to COVID-19 has not only slowed the spread of the virus, but may also continue to impact TB trends. Our research thus forecasts a continued decline in TB incidence, aligning with the WHO's objective of eliminating TB in the future [11].

The implementation of nationwide strategies such as city closures at the onset of the COVID-19 pandemic in early 2020 led to an increase in vulnerability to TB and a reduction in travel restrictions, population mobility, and TB notification rates [18,25]. Subsequent policies aimed at easing restrictions on population mobility from 2021 to 2022 may have mitigated the impact of COVID-19 interventions on TB compared to 2020. While the TB incidence decreased during the COVID-19 outbreak due to certain containment measures, potential factors such as poverty, increased household exposure to TB infection, susceptibility, and drug resistance could have offset these positive effects [3].

The potential impact of COVID-19-related interventions on the reduction in TB notifications can be explained by several factors [4,26–29]. One might be the disruption of medical services, where strict traffic control measures and activity restrictions during the pandemic may have led to interruptions in the supply of drugs and laboratory equipment in medical institutions [4,27–29]. Another factor could be resource limitations, whereby the flow and allocation of resources may have been constrained during the health care system overload, and whereby hospitals might prioritise emergencies, reallocating medical staff and diagnostic platforms for COVID-19 treatment, thereby reducing the focus on TB detection and treatment [4,27–29]. This could have also been related to challenges in service provision; providing services in high-burden environments can be challenging, especially when there are similarities in clinical symptoms between TB and COVID-19 [4]. Fear of contracting COVID-19 may have also deterred individuals from seeking medical care, further impacting TB diagnosis and treatment [4,27]. We should also consider the role of travel restrictions, as requirements for a COVID-19 nucleic acid-negative certificate for travel may have discouraged individuals, including TB patients, from seeking medical assistance. Reduced hospital visits could have been another relevant factor; to alleviate hospital congestion, individuals with chronic diseases or mild symptoms may have been advised against seeking medical help, potentially delaying TB diagnosis and treatment [4]. Lastly, we can also consider the effect of limited access to health care: shortened operating hours of health facilities and decreased ability of vulnerable populations (such as the elderly or those with mobility challenges) to afford care or transportation can hinder access to health care services for TB patients [4,26,28,29]. Research suggests that, while the stringent response to COVID-19 has been effective in curbing virus transmission, it may have inadvertently impacted TB control programs [29]. Therefore, it is essential to prioritise interventions that ensure the continuity of essential TB preventive services, guarantee adequate treatment for diagnosed TB patients, and address the vulnerabilities exacerbated during the pandemic [30]. This proactive approach is crucial in maintaining progress in TB control efforts amidst the challenges posed by the COVID-19 pandemic.

TB demonstrated a continued decline over the period analysed in our study, with an AAPC of –7.3. The downward trend in TB cases during 2018–21 (APC = –11.4) was more pronounced than that observed in 2013–18 (APC = –4.7). This may have been related to effective government measures, increased technical proficiency among health care professionals, standardised diagnosis and treatment protocols for TB patients, intensified TB prevention and control efforts targeting key groups and areas, improved living standards, increased health awareness among the population, and restricted mobility of high-risk populations due to the COVID-19 outbreak [3]. The more intense decline in TB cases during 2018–21 compared to 2013–18 could be influenced by the decrease in TB reports resulting from the impact of COVID-19. Our findings also suggest a future downward trajectory in the TB epidemic, aligning with the WHO's objective of eliminating TB [11].

Several factors can influence the prediction of TB trends, including seasonality, demographic shifts, regional disparities, and data quality and availability [31]. Temperature variations and seasonal patterns also contribute to shaping TB trends [32]. It is crucial to emphasise the risks associated with delayed TB diagnosis during epidemics. Delays in medical care seeking, diagnosis, and treatment, as well as inadequate management of TB patients, can exacerbate disease progression, elevate morbidity and mortality risks, and heighten the potential for TB transmission within communities and households, hindering elimination efforts. Despite current interventions, the reduction achieved falls short of meeting the WHO's goals at various junctures. In recent years, some countries have experienced a gradual decline in TB incidence or heightened risks of recurrence due to factors such as climate change, significant population movements, rising drug-resistant TB cases, ageing populations, poverty, malnutrition, substance abuse, HIV co-infection, diabetes, hypertension, and other prevalent conditions [3]. Comprehensive intervention strategies addressing these multifaceted challenges are currently needed to expedite the elimination of TB.

TB exhibits marked seasonality, typically peaking in spring, as corroborated by research conducted in the USA [33], Mongolia [34], and India [35], particularly during March or April. This seasonal trend coincides with dry weather and lower temperatures – conditions that facilitate the rapid spread of the disease. However, the seasonal patterns of TB incidence we observed here differ from those reported in various regions. For instance, the peak incidence in the UK [36] and Hong Kong [37] occurs exclusively in the summer, while studies in Spain [38] and South Africa [39] indicate that TB seasonality peaks in late spring or early summer. In contrast, Tunisia experiences TB peaks in winter and spring [40]. Several factors may contribute to these seasonal variations, the first being natural environmental factors. Social factors could also play a crucial role. For example, the Chinese New Year typically falls in January or February, during which there is a noticeable decline in the number of individuals seeking medical attention. Consequently, patients may delay visiting health care providers until March or April, resulting in increased reported cases – a phenomenon unique to China known as the ‘Spring Festival effect’ [4]. The irregular lifestyle patterns during the Spring Festival, followed by a return to work, may contribute to this delay. Additionally, considerable temperature fluctuations in spring may increase human susceptibility to TB, further exacerbating the situation. Delays in TB diagnosis can also lead to an uptick in reported cases during spring. Some studies suggest that a low point in vitamin D levels at the end of winter may reactivate latent TB infections [41]. Another hypothesis posits that increased TB transmission in spring may stem from indoor crowding during the winter months, facilitating greater exposure [42]. Understanding these seasonal factors influencing TB can provide insights into the disease's pathogenesis and inform new treatment approaches. Therefore, it is essential to implement effective TB prevention and control measures during the spring. Recommended strategies include proper mask-wearing and timely medical consultations to mitigate the risk of TB epidemic [26].

This study has potential limitations. First, while the BSTS model can deliver reliable predictions even with small sample sizes, capturing the underlying patterns in the data necessitates sufficient and comprehensive foundational data. Second, passive monitoring systems may experience challenges such as delayed reporting, missed cases, false positives, or inadequate diagnoses. Future applications of the BSTS model should account for these factors and incorporate new data to refine the model parameters for optimisation. Third, it is important to note that as the number of forecasted steps increases, the model's performance may decline. Fourth, TB transmission is influenced by various complex factors. Therefore, utilising the BSTS model for predictions can be improved by integrating additional factors, such as meteorological conditions and air quality indicators. Finally, we were unable to collect more detailed data on age, gender, HIV, and rural and urban areas through the reporting system for notifiable infectious diseases, which limited our analysis.

## CONCLUSIONS

Our findings show that interventions associated with the COVID-19 pandemic have had significant medium- and long-term effects in reducing the incidence of TB. The data obtained from Henan indicate a declining trend in overall TB incidence, characterised by distinct seasonal and cyclical patterns, which reinforces the necessity for comprehensive surveillance and responsive public health strategies. The use of BSTS model in our study has proven invaluable not only for forecasting TB epidemic trends but also for assessing the causal influence of policy implementations on TB incidence reduction during the pandemic. As the global health community seeks to mitigate the impacts borne from the COVID-19 pandemic, the experiences drawn from this period must inform and shape future TB control efforts. By leveraging the lessons learned, stakeholders can work collaboratively towards achieving the critical goal of a TB-free world by 2035, ensuring that the health systems are better prepared to confront any future public health challenges.
